#  The effects of fixational eye movements on population responses in V1. Keynote at the 20th European Conference on Eye Movements in Alicante, September 22, 2019 

**DOI:** 10.16910/jemr.12.7.7

**Published:** 2019-11-25

**Authors:** Hamutal Slovin

**Affiliations:** Bar-Ilan University. Ramat Gan, Israel

**Keywords:** V1, fixational eye movements, population response, microsaccades

## Abstract

During visual fixation, the eyes make small and fast movements known as microsaccades (MSs). The effects of MSs on neural activity in the visual cortex are not well understood. Utilizing voltage-sensitive dye imaging, we imaged the spatiotemporal patterns of neuronal responses induced by MSs in early visual cortices of behaving monkeys.

Our results reveal a continuous “visual instability” during fixation: while the visual stimulus moves over the retina with each MS, the neuronal activity in V1 ‘hops’ within the retinotopic map, as dictated by the MS parameters. Neuronal modulations induced by MSs are characterized by neural suppression followed by neural enhancement and increased synchronization. The suppressed activity may underlie the suppressed perception during MSs whereas the late enhancement may facilitate the processing of new incoming image information. Moreover, the instability induced by MSs applies also to neural correlates of visual perception processes such as figure-ground (FG) segregation, which appear to develop faster after fixational saccades.

**Video stream:** https://vimeo.com/362367119

